# Assessment of the Impact of Decellularization Methods on Mechanical Properties of Biocomposites Used as Skin Substitute

**DOI:** 10.3390/ma14174785

**Published:** 2021-08-24

**Authors:** Bożena Gzik-Zroska, Kamil Joszko, Wojciech Wolański, Sławomir Suchoń, Michał Burkacki, Marek Ples, Jerzy Malachowski, Michał Tomaszewski, Arkadiusz Szarek, Grzegorz Stradomski, Diana Kitala, Mohsen Akbari, Marek Gzik

**Affiliations:** 1 Department of Biomaterials and Medical Devices Engineering, Faculty of Biomedical Engineering, Silesian University of Technology, Roosevelta 40 Str., 41-800 Zabrze, Poland; bozena.gzikzroska@polsl.pl; 2 Department of Biomechatronics, Faculty of Biomedical Engineering, Silesian University of Technology, Roosevelta 40 Str., 41-800 Zabrze, Poland; wojciech.wolanski@polsl.pl (W.W.); slawomir.suchon@polsl.pl (S.S.); michal.burkacki@polsl.pl (M.B.); marek.ples@polsl.pl (M.P.); marek.gzik@polsl.pl (M.G.); 3 Faculty of Mechanical Engineering, Institute of Mechanics and Computational Engineering, Military University of Technology, Gen. Sylwestra Kaliskiego 2, 00-908 Warszaw, Poland; jerzy.malachowski@wat.edu.pl (J.M.); michal.tomaszewski@wat.edu.pl (M.T.); 4 Department of Technology and Automation, Faculty of Mechanical Engineering and Computer Science, Czestochowa University of Technology, 21 Armii Krajowej Av., 42-201 Czestochowa, Poland; arek@iop.pcz.pl; 5 Faculty of Production Engineering and Materials Technology, Czestochowa University of Technology, 19 Armii Krajowej Av., 42-201 Czestochowa, Poland; gstradomski@wip.pcz.pl; 6 Stanislaw Sakiel Burn Treatment Centre, Jana Pawla II 2 Str., 41-100 Siemianowice Slaskie, Poland; diana.kitala@clo.com.pl; 7 Laboratory for Innovations in Microengineering (LiME), Department of Mechanical Engineering, University of Victoria, Victoria, BC V8P 5C2, Canada; makbari@uvic.ca; 8 Centre for Advanced Materials and Related Technologies (CAMTEC), University of Victoria, Victoria, BC V8P 5C2, Canada; 9 Biotechnology Center, Silesian University of Technology, Akademicka 2A, 44-100 Gliwice, Poland

**Keywords:** decellularization, skin substitute, mechanical properties

## Abstract

This work aimed to assess the impact of acellularization and sterilization methods on the mechanical properties of biocomposites used as a skin substitute. On the basis of the statistical analysis, it was ascertained that the values of the Young modulus for the samples before the sterilization process—only in the cases of substances such as: trypsin, 15% glycerol and dispase—changed in a statistically significant way. In the case of dispase, the Young modulus value before the sterilization process amounted to 66.6 MPa, for trypsin this value equalled 33.9 MPa, whereas for 15% glycerol it was 11 MPa. In the case of samples after the completion of the sterilization process, the analysis did not show any statistically significant differences between the obtained results of Young’s modulus depending on the respective reagents applied. It was confirmed that different methods of acellularization and the process of sterilization effect the alteration of mechanical properties of allogeneic skins. In the case of the decellularization method using SDS (Sodium Dodecyl Sulfate), liquid nitrogen and 85% glycerol the highest values of strain were observed. In the authors’ opinion, it is the above-mentioned methods that should be recommended in the process of preparation of skin substitutes.

## 1. Introduction

Skin substitutes are an effect of a complex technological process and should be treated as composite-like constructs. They are usually built from a component playing the role of a load-bearing structure as well as different types of cells. The creation of such a structure is a complex and elaborate issue. Nowadays, the most popular treatment of the defect or loss of skin layers involves the use of biostatic grafts (deprived of living cells). They are often called biological dressings or scaffolds and constitute the above-mentioned load-bearing structure.

Skin is an organ of a complex, stratified structure that is particularly vulnerable to different defects and damage, especially through immediate contact with the external environment [1]. Despite relatively big—as for mammals—regenerative abilities, there occur injuries which the organism finds impossible to repair and self-regenerate [2,3,4,5]. Due to this fact, the replacement of damaged skin fragments is a crucial and often decisive aspect of the treatment [6].

Skin grafts are now the best way of treatment of bigger defects and losses within this organ, and with the application of proper clinical procedures they do not pose a high operative and post-operative risk. Unfortunately, the availability of the graft material is limited by various factors [7].

Due to the insufficient access to graft materials, since the late 19th century research has been carried out on the methods of obtainment of suitable materials to be used as skin substitutes, i.e., utilizing tissue engineering [8].

The preparation process of scaffolds involves acellularization (removal of cells), sterilization and storage in proper conditions. The first stage of the creation of a biological dressing is the removal of the donor’s cells with the preservation of the structure of the extracellular matrix and the sterilization of the biological material. There are many methods of cell removal (in other words: acellularization or decellularization), including: chemical, biological, and mixed procedures [9,10,11,12].

The selection of the most suitable measure of the removal of cells from tissues or organs depends on various factors, such as tissue cellularity, density, lipid content, or structure thickness. What should be borne in mind is the fact that each method will cause modifications in the content of the extracellular matrix and some disturbance in the ultrastructure, which may have an impact, among other things, on the change of mechanical properties.

The literature includes works concerned with the determination of mechanical properties of the skin and the effect of sterilization on the change of mechanical parameters of materials used in interventional surgery [13,14,15,16,17,18,19,20]. In the literature, the tensile properties of ex vivo native skin samples have been studied by Nì Annaidh et al. [4], who calculated engineering stresses and strains and the elastic modulus for different back areas and different orientations. They demonstrated the variability of tissue properties in relation to specimen orientation (referred to as Langer lines). Yoder and Elliott [21] performed static tensile tests on various soft tissue materials, emphasizing the importance of the mechanical properties of these tissues for the success of surgical procedures. It is also proposed that acellular dermal matrix allograft increases keratinized soft tissue in the dental peri-implant area [22].

The skin grafts generated in the above-described way are often damaged during the transplantation procedure, which may be caused by the deterioration of their mechanical properties as a result of decellularization [23]. There are also many mentions in the literature concerning the analysis of mechanical properties of various biological structures, which were later used in the process of numerical modeling [24,25,26,27,28]. However, there is a lack of literature on the assessment of mechanical properties of scaffolds (obtained from donors) subjected to the process of decellularization and sterilization. It seems necessary to conduct research, which would define the impact of decellularization methods (most common ones) on the alteration of mechanical properties of graft. This research aims to assess decellularization methods of the matrices of biological scaffolds used as biological dressings by comparing their mechanical properties. The results of the investigations may enable the selection of the most effective decellularization method taking into account the assessment of the most suitable mechanical properties from the perspective of the transplantation procedure.

## 2. Materials and Methods

### 2.1. Specimens Preparation

**Figure 1:**
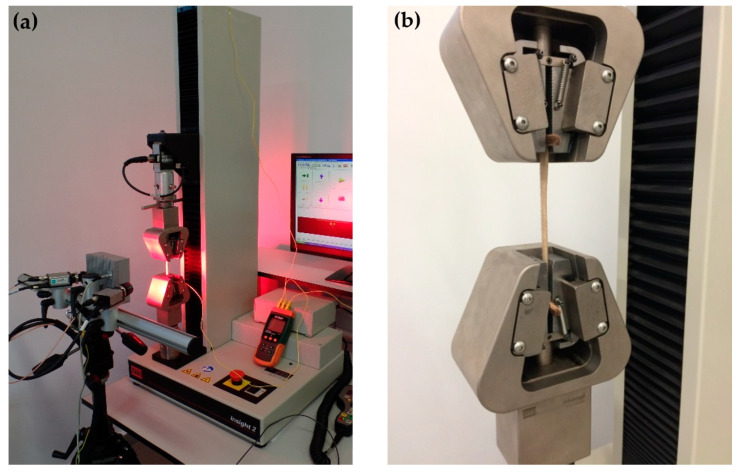
(**a**) strength testing machine; (**b**) a method of fixation of a sample in the clamps.

The tests were conducted by means of a strength testing machine MTS Insight 2 machine (MTS Systems, Eden Prairie, MN, USA) (Figure 1). In total, 80 samples (10 for each method) from three different donors were tested. Mechanical properties of the skin depend on the direction of excision of the sample in relation to Langer’s lines and the system of collagen fibres [3,4,20]. Taking the above into consideration, the samples were excised in the form of a dermatome, parallel to Langer’s lines, from the donor’s back. This way of cutting ensures a uniform layout of collagen fibres and excludes their impact on mechanical properties measured in this work. After taking the samples, they were stored at a temperature of −80 °C. The samples were subjected to the procedure of cell removal following methods outlined below:

1. Chemical procedure (0.1% SDS—Sodium Dodecyl Sulfate, 3% Triton X-100)
2. Enzymatic procedure (0.5% Trypsin, 2.4 U/mL Dispase)
3. Physical procedure (Liquid nitrogen −196 °C for 4 h)
4. Mixed procedure (in two stages: first is enzymatic procedure, and then chemical procedure, both as described above)

A part of the samples was subjected to the incubation procedure in 15% and 85% glycerol. The specimens were stored at a temperature of −20 °C; prior to mechanical tests, samples were thawed in saline at room temperature of 21 °C for 20 min. A detailed description of the preparation of the samples for testing was presented in the publication [13].

### 2.2. Uniaxial Tensile Testing

Prior to testing, it was necessary to prepare the strength testing machine MTS Insight 2 machine (MTS Systems, Eden Prairie, MN, USA). In order to perform this, special clamps were mounted on the machine to enable proper fixation of the sample. Before placing the sample in the clamps, it was necessary to wrap its endings around specially prepared small rollers, which prevented spontaneous slipping of the specimen from the clamps. In addition, it eliminated an immediate pressure exerted on the sample and decreased the concentration of stresses at the place of the sample’s fixation. Prior to testing, measurements of the thickness (0.24 ± 0.03 mm) and width (18 ± 1.92 mm) of the samples were conducted using a certified thickness gauge and a slide caliper, respectively. In addition, before the performance of the test, the initial distance between clamps was measured which was 60 mm. Next, the sample was stretched in the direction of the longitudinal axis (Figure 1) in quasi-static conditions at a velocity of 5 mm/min [4,14]. The results were recorded at a frequency of 10 Hz.

**Figure 2:**
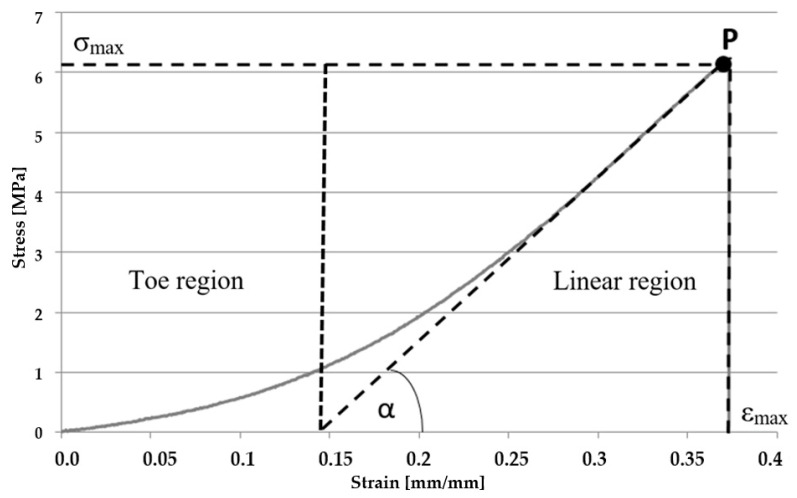
Example of the graph of stress in the strain function for the samples tensile.

The temperature of storage of samples has a significant influence on the change of their mechanical parameters [24,25]. The gradient of the stress-strain curve is decreasing along with the increase in temperature. This fact is caused by the stretching and sliding of collagen particles in the collagen fibre network. With the increase in temperature, an organized collagen structure changes into randomly positioned particles resulting in decreasing stiffness. That is the reason why all samples, within the framework of this research, were stored at a temperature of −20 °C for a period of 2 months from the moment of their sampling. Prior to testing, the samples were defrosted in a physiological salt solution at a room temperature of 21 °C over a period of 20 min. The same procedure is applied before the implantation process in the operational theatre. As a result of the conducted tests, the following parameters were determined: Young’s modulus, which was calculated on the basis of the graph of stress in the function of strain in a proportional scope. The value of the Young modulus equals the tangent of the inclination angle of the segment proportional to the axis of strain. The example of such a diagram is presented in (Figure 2). The presented graph depicts two areas of toe region and linear region, such as in [24], and is very similar to the periodontal biline characteristics. In the case of the analyzed material, toe region constitutes around 1/3 of the total scope of strain which reflects the viscoelastic properties of biological material. With regards to this range, mainly elastin fibres are responsible for the deformation, while collagen fibres stand for the region analyzed by the authors. It was assumed that the mechanical properties of the tested material were determined for the linear region, which represents a model of a linearly elastic material. The value of the maximum stress σ_max_ and maximum strain ε_max_ were read at point ‘P’ for the linear region, which is marked in Figure 2.

## 3. Results

### 3.1. Data Obtained in Experimental Tests

**Figure 3:**
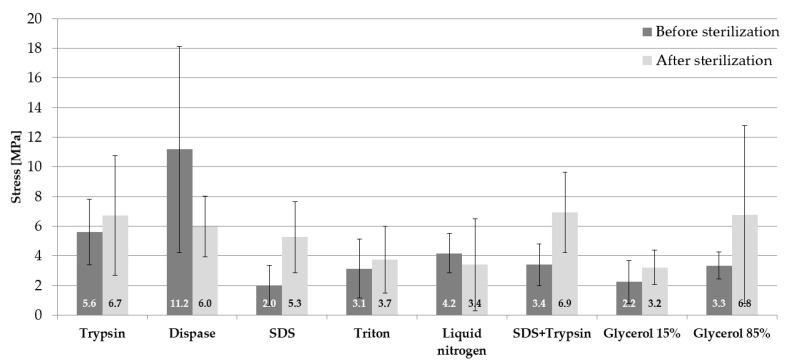
Mean values of maximum stress.

The obtained test results of mean stress values along with standard deviations (SD) for the samples before and after sterilization are presented in Figure 3. The highest mean stress values for the samples before sterilization were observed for the samples subjected to decellularization by means of dispase 11.2 ± 6.9 MPa. The next substance which conditions high values of stress is trypsin 5.6 ± 2.2 MPa. Other substances have similar values of stress (within the range of 2–4.2 MPa). In the case of samples subjected to the process of sterilization, the highest mean stress values were observed for three methods of cell removal: SDS and trypsin 6.9 ± 2.6 MPa, 85% glycerol 6.8 ± 5.9 MPa, and trypsin 6.7 ± 4.1 MPa. However, it was noticed in relation to the samples without sterilization that the process of sterilization caused an increase in mean stresses for almost all substances. The exceptions are as follows: dispase, where there was a decrease in stress value by half, and liquid nitrogen, where the differences were really small 0.8 MPa.

**Figure 4:**
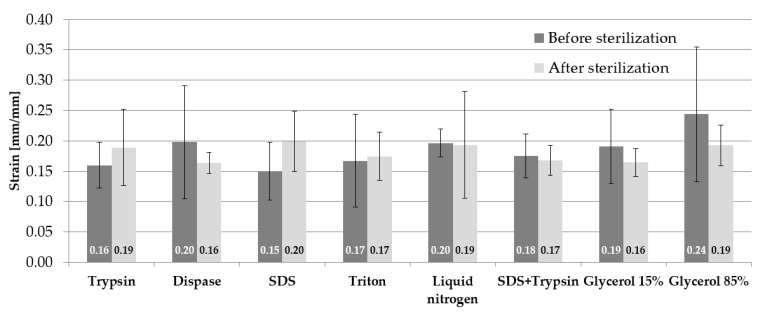
Mean values of maximum strain.

The obtained strain mean values with standard deviations (SD) for the samples before and after sterilization are juxtaposed in Figure 4. The highest mean strain for the samples before sterilization occurs for the samples subjected to incubation in 85% glycerol 0.24 ± 0.11 mm/mm, whereas the lowest strain value occurred in the case of applied SDS 0.15 ± 0.05 mm/mm. The values of the mean strain in the case of samples subjected to sterilization are similar for three substances: trypsin, liquid nitrogen, and 85% glycerol amounting to 0.19 mm/mm. The highest mean strain value was observed in the case of SDS 0.20 ± 0.05 mm/mm, whereas the lowest was in the case of dispase and 15% glycerol 0.16 ± 0.02 mm/mm. The application of the sterilization process influences the decrease in mean strain values for dispase by 0.04 mm/mm, liquid nitrogen by 0.01 mm/mm, SDS and trypsin by 0.01 mm/mm, 15% glycerol by 0.03 mm/mm, and 85% glycerol by 0.05 mm/mm.

**Figure 5:**
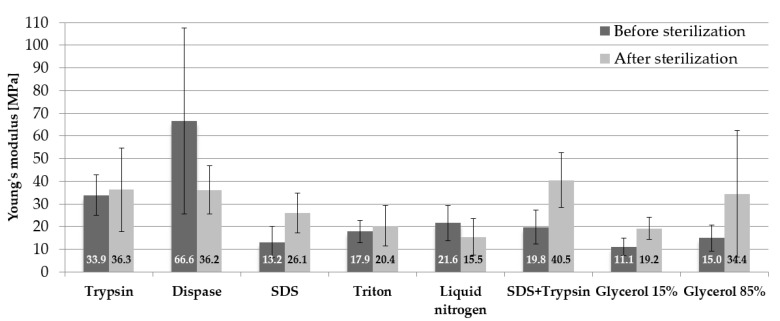
Mean values of Young’s modulus.

The obtained results of Young’s modulus mean values along with standard deviations (SD) in relation to the samples before and after sterilization are presented in Figure 5. In terms of the samples before sterilization, the use of dispase favourable affected mechanical properties. The tested samples were characterised by the highest values of the mean modulus of elasticity 66.6 ± 40.9 MPa. Cryopreservation in 15% glycerol was responsible for the fact that the tested specimens obtained the lowest values of Young’s modulus 11.1 ± 3.9 MPa.

In regards to the specimens subjected to sterilization, the use of the mixed procedure (SDS and trypsin) led to the situation where the values of Young’s modulus were the highest 40.5 ± 12.1 MPa. Decellularization involving the use of liquid nitrogen reduced the value of Young’s modulus by twice (about 25 MPa). The use of sterilization led to a slight increase in the mean Young’s modulus in relation to trypsin (a difference of 2.4 MPa) and triton (a difference of 2.5 MPa) and to nearly a double increase in the value of Young’s modulus in relation to SDS, SDS + trypsin, 15% glycerol and 85% glycerol. Radiation sterilization reduced the value of Young’s modulus by 30.4 MPa in relation to dispase and by 6.1 MPa in relation to liquid nitrogen.

### 3.2. Statistical Analysis

The assessment and interpretation of the results involved the performance of statistical analysis using the Statistica software.

#### 3.2.1. Data Analysis: Reagent, Sterilization and Strain

The strain-related statistical analysis involved the performance of the multi-factor analysis of variance (ANOVA). The aforesaid analysis aimed to determine (i) whether there were significant differences in the obtained values of strains resulting from the application of a given reagent (ii), whether there were significant differences in the obtained values of strains resulting from the performance of sterilization, and (iii) whether there was an interaction between such factors as the reagent and sterilization.

The performance of the statistical analysis was preceded by the verification of all ANOVA test-related assumptions including the following:

1. Independence of random variables in populations (groups) under consideration
2. Measurability of analyzed variables
3. Normality of the distribution of variables in each population (group)
4. Homogeneity of variance in all populations (groups)

**Figure 6:**
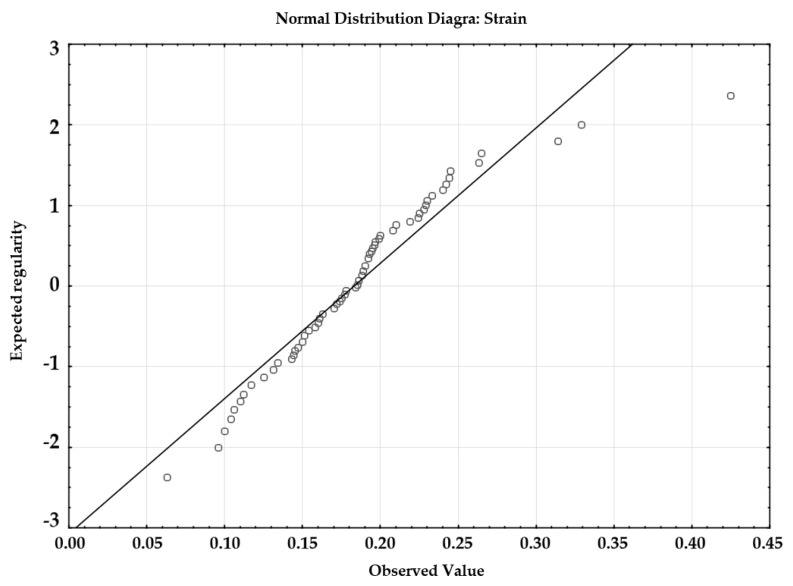
Distribution of the normality of strains in the analyzed samples.

The variables are measurable and independent, therefore, the first two assumptions are satisfied. The normality of the distribution of variables has been verified using the diagram of normality (Figure 6).

Figure 6 reveals that the distribution of the values subjected to analysis was normal. The assumption related to the homogeneity of variance was verified using the Brown-Forsythe test, because, in relation to the unequal sizes of groups, it provides better results.

The analysis of variance based on the above-named method is performed on the basis of absolute deviations from the median of the group. At the first stage, the following research hypotheses (Zero Hypothesis (H_0_) and Alternative Hypothesis (H_1_)) were formulated:

**Hypothesis** **0** **(H_0_).** *Variance of the dependent variable in the groups of the factor was the same*.

This is example 1 of an equation:

(1) $$H_{0}=\delta_{1\;}^{2}=\delta_{2}^{2}=\delta_{3}^{2},$$

**Hypothesis** **1** **(H_1_).** *The Variance of the dependent variable in the groups of the factor. The variable assumed as the first was the strain, whereas the grouping variable was the reagent (Table 1)*.

The performance of the Brown-Forsythe test revealed the condition of the homogeneity of variance was satisfied *p* = 0.778 (*p* > 0.05). Afterwards, the assumption of the homogeneity of variance was verified using the Brown–Forsythe test, where the strain was adopted as the dependent variable and sterilization was adopted as the grouping variable (Table 2).

In relation to the analysis where the grouping variable was sterilization, the condition of the homogeneity of variance was also satisfied: *p* = 0.171 (*p* > 0.05). After the verification of all of the assumptions related to the analysis of variance, it was possible to perform the statistical analysis, the results of which are presented in Table 3.

Table 3 reveals that:

- there are no significant differences in the obtained strain-related values resulting from the reagent *p* = 0/659, (*p* > α, α = 0.05);
- there are no significant differences in the obtained strain-related values resulting from the performance of sterilization *p* = 0.748, (*p* > α, α = 0.05);
- there is no interaction between the factors “reagent” and “sterilization” *p* = 0.631, (*p* > α, α = 0.05).

**Figure 7:**
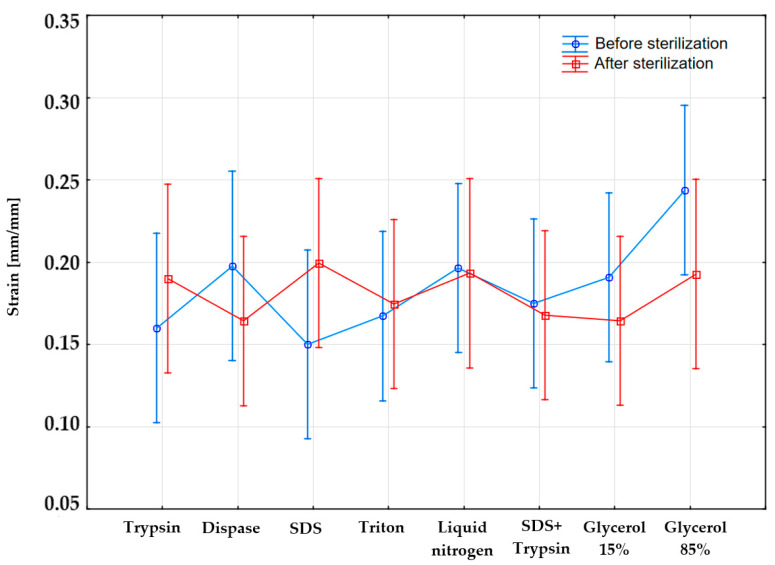
Graphic interpretation of the interaction effect.

In addition, the study involved the making of a diagram of interaction (Figure 7) between the factors, i.e., “reagent” and “sterilization”, the curves of which overlapped, implying the lack of dependence between the reagent and sterilization.

#### 3.2.2. Data Analysis: Reagent, Sterilization and Young’s Modulus

**Figure 8:**
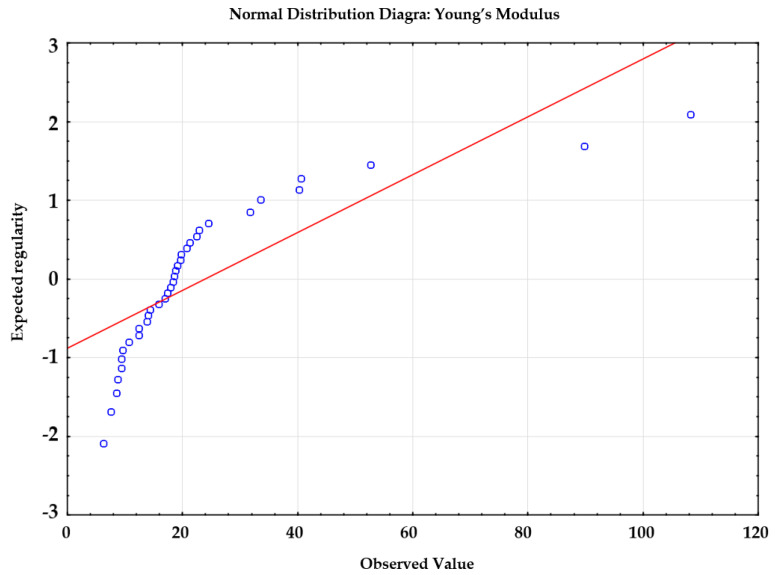
Graphic interpretation of the interaction effect.

The statistical analysis was performed to determine whether the manner of decellularization and storage of samples affected the obtained values of Young’s modulus. To this end, it was necessary to apply the multi-purpose analysis of variance (ANOVA). Similar to the analysis concerning the value of strain, it was necessary to verify the satisfaction of all test-related assumptions. The assumption of the independence and measurability of the analyzed variables was satisfied. Figure 8 reveals that the analyzed values did not undergo normal distribution because the points do not lie along the distribution function being the straight line.

For this reason, the value of Young’s modulus was compared using a non-parametric test. The analysis involving the use of the non-parametric test is possible even if the assumptions related to parametric tests have not been satisfied. The test used for this purpose was the Kruskal–Wallis test, which compares several independent groups. The test aims to determine the measures of orientation (distribution) of a tested feature in groups subjected to comparison. The adopted hypotheses were the following:

- zero hypothesis (H_0_), assuming that the applied substance does not affect the obtained values of the modulus of elasticity,
- alternative hypothesis (H_1_), assuming the existence of statistically significant differences between substances under consideration in terms of the obtained values of Young’s modulus.

The above-named test is based on ranks of observation, which means that if all samples come from one population, it is possible to expect that the mean ranks in individual groups will be similar.

The test statistics is expressed with the following formula:

(2) $$T=\frac{12}{n\left(n+1\right)}\sum_{i=1}^{k}\frac{R_{i}^{2}}{n_{i}}-3\left(n+1\right),$$

where:

k—number of samples,
$R_{i}$—sum of ranks in the *i*th group,
$n_{i}$—size of the *i*th group,
n—total size of all groups.

##### Samples before Sterilization

The analysis was performed in order to identify the independent (grouping) variable, i.e., the type of applied reagent and the dependent variable, i.e., Young’s modulus. Table 4 presents the results of the statistical analysis. The table contains data related to codes assigned to individual substances, numbers of tested samples in individual groups, as well as the values of sums and mean ranks.

The test statistics is value H (also designated as K). The higher value H is, the greater the difference between ranks in relation to tested groups. In this analysis, the value of the Kruskal–Wallis test was H = 18.83, whereas the significance level *p* = 0.0087. For this reason, it is possible to reject the zero hypothesis and adopt the alternative one. The above-presented results (at significance level *p* = 0.0087, *p* < α) justify the conclusion that there is at least one pair of reagents significantly diversified in relation to obtained values of Young’s modulus. The determination of statistically significant differences (if any) between the substances involved the performance of repeated (multiple) comparative tests adopting the following hypotheses: zero hypothesis, assuming the lack of statistically significant differences between the substances under consideration

H_0_:M_1_ = M_7_ (3)

and alternative hypothesis, assuming the existence of statistically significant differences between the substances under consideration

H_1_:M_1_ ≠ M_7_ (4)

The tests were performed adopting significance level α = 0.05.

The procedure of multiple (repeated) comparisons in the Kruskal-Wallis test was performed in the manner presented below.

The first stage involved the calculation of the mean values of ranks in relation to the tested population using the following formula:

(5) $$\overset{´}{R_{i}}=\frac{R_{i}}{n_{i}}$$

where:

$R_{i}$—sum of ranks in relation to a given group,
$n_{i}$—number of observations in a group subjected to consideration.

The critical value, i.e., the equivalent to LSD (least significant differences) is calculated using the following formula:

(6) $$D=\chi_{\alpha ;k-1}^{2}\sqrt{\left[\frac{n\left(n+1\right)}{12}\right]\left(\frac{1}{n_{i}}+\frac{1}{n_{j}}\right)}$$

where:

$\chi^{2}$—critical value for the chi-square test,
k—number of groups subjected to comparison,
n—total number of observations,
$n_{i}$, $n_{j}$—sizes (in terms of numbers) of groups subjected to comparison.

where the absolute value of the difference of ranks

(7) $$D=\left|{\overset{´}{R}}_{i}-{\overset{´}{R}}_{j}\right|$$

is higher than *D*, the groups subjected to comparison are recognized as statistically significantly different.

The repeated (multiple) tests (Table 5) revealed that, in relation to substance 1 (trypsin) and 7 (15% glycerol), probability level *p* = 0.03, therefore, *p* < α. As a result, the zero hypothesis should be rejected and the alternative one should be accepted. There are statistically significant differences between substance 1 (trypsin) and 7 (15% glycerol). As regards reagent 2 (dispase) and 7 (15% glycerol), probability level *p* = 0.04 (*p* < α), which means that also in this case it is necessary to reject the zero hypothesis and adopt the alternative one. There are statistically significant differences between substance 2 (dispase) and 7 (15% glycerol). In terms of the remaining methods, *p* > α, therefore, there are no grounds for the rejection of the zero hypothesis. There are no statistically significant differences between the remaining substances.

**Figure 9:**
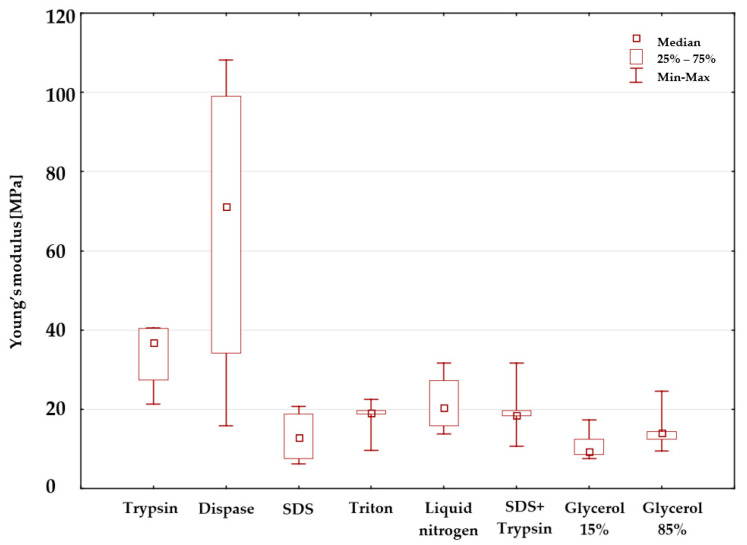
Diagram presenting the mean value and the standard deviations of Young’s modulus in relation to human samples before sterilization performed using various substances.

Figure 9 presents the graphic interpretation of the results. The analysis of the diagram reveals significantly higher values of the mean Young’s modulus in relation to substances, i.e., dispase (approximately 70 MPa) and trypsin (approximately 38 MPa) in comparison with the remaining reagents (approximately 15 MPa). The lowest value was obtained for 15% glycerol. The diagram confirms the results of repeated (multiple) comparisons, i.e., statistically significant differences between dispase and 15% glycerol, as well as trypsin and 15% glycerol.

##### Samples after Sterilization

The statistical analysis based on the data obtained from the sample after sterilization was also performed using the Kruskal–Wallis test (Table 6). The independent (grouping) variable was the type of applied substance, whereas the dependent variable was the value of the modulus of elasticity.

Due to the fact that the test result was H = 15.29 and *p* = 0.0325 (*p* < α), the zero hypothesis should be rejected and the alternative one should be accepted. It can be concluded that there are statistically significant differences in the obtained results concerning Young’s modulus in relation to applied substances.

The determination of the reason for rejecting the zero hypothesis required the use of repeated (multiple) comparisons, the results of which are presented in Table 7.

As the results of the repeated comparisons present probability level *p* > α, there are no grounds for rejecting the zero hypothesis. At an established significance level α = 0.05 it is difficult to determine specifically which groups of reagents differ in terms of Young’s modulus. As a result, it should be assumed that there are no statistically significant differences between the substances under consideration.

## 4. Discussion

Where the spontaneous healing of a burn wound is impossible or ineffective because of the significant advantage of scar formation processes, surgical procedures are applied (usually in the cases of deep, i.e., 3rd and 4th degree burns) involving the removal of necrotic tissues followed by the covering of the wound with the most appropriate biological dressing. The treatment aims to provide the fastest, most effective, functional and morphological reconstruction of the skin damaged by burning. The protection of the microenvironment of the wound against the external environment is provided by dressings used for the nursing of burn wounds. The aforesaid dressings are characterised by a high level of regeneration of damaged tissues, providing protection against secondary mechanical injuries and the colonization of microorganisms. The primary and, at the same time, the most favourable method of treating burn wounds is autologous grafts, the undoubted advantages of which include nearly immediate availability and the long-term covering of wounds. On the other hand, using the patient’s own implants entails pain in the area out of which a given graft was taken. In addition, the applicability of the aforesaid method is limited in patients with vast burn wounds [5]. For this reason, dressings are often taken from donors. The assessment of the mechanical properties of skin is of the essence both for reconstructive and plastic surgery. Proper mechanical properties of dressings obtained from skins of transgenic pigs constitute an important element as regards the operative treatment of burn wounds. Opinions expressed by the surgeons from the Centre of Burns Treatment indicate plastic and reconstructive procedures are accompanied by cases of damage to implanted skin dressings. Such situations result from the preparation of dressings, significantly affecting their mechanical properties. For this reason, it is important to ensure the most favourable material parameters of the skin following the process of decellularization and sterilization. The optimal decellularization treatment should minimize cellular and nuclear materials in the matrix, without affecting the integrity of the tissue structure, and consequently its mechanical properties. Generally speaking, process decellularization cannot adversely affect or influence collagen, elastin, and extracellular components. The aforesaid issue inspired the authors of this publication to investigate the issue concerning the effect of decellularization methods and sterilization on the mechanical properties of dressings.

The statistical analysis of the results obtained in related tests revealed that the value of Young’s modulus before sterilization changed in a statistically significant manner only in relation to trypsin, 15% glycerol, and dispase. In terms of dispase, the values of Young’s modulus before sterilization amounted to 66.6 [MPa]. In relation to trypsin, the aforesaid value amounted to 33.9 [MPa], whereas in 15% glycerol—11 [MPa]. In regards to the samples after sterilization, the analysis did not reveal statistically significant differences between the obtained values of Young’s modulus in relation to a given reagent. The obtained values of Young’s modulus and stress at break are on a similar level as in the scientific works of other authors [4,21,29].

In regards to the surgical procedures, dressings after decellularization and sterilization should be characterized by the highest possible strain values. Skin dressings made using the SDS methods, liquid nitrogen and 85% glycerol, were characterised by high strain values, i.e., SDS—0.2 [mm/mm], liquid nitrogen—0.19 [mm/mm] and 85% glycerol—0.19 [mm/mm]. The above-presented mechanical parameters are required in plastic and reconstructive surgery as such dressings are less damageable during procedures.

## 5. Conclusions

The research involved tests of the uniaxial stretching of human skin samples. In total, 80 samples were tested using eight methods of decellularization. Half of the tested samples were subjected to the process of radiation sterilization (35 kGy) performed in compliance with the procedures of the Tissue Bank. On the basis of the conducted investigations, it was ascertained that different methods of decellularization and the sterilization process affected the mechanical properties of the skin samples. However, the changes are not statistically significant. The highest strain values were observed in relation to decellularization methods involving the use of SDS, liquid nitrogen, and 85% glycerol. In the opinion of the authors, due to the most advantageous mechanical properties, these methods could be recommended in the preparation of allogeneic skin dressings. The above-mentioned methods should ensure a lower probability of damage during surgical procedures.

## Figures and Tables

**Table 1:** Verification of the assumption of the homogeneity of variance by means of the Brown-Forsythe test with the reagent adopted as the grouping variable.

	Brown-Forsythe Variance Homogeneity Test Marked Effects Are Relevant with *p* < 0.05							
Variable	SS Effect	df Effect	MS Effect	SS Error	df Error	MS Error	F	*p*
Strain	0.006	7	0.001	0.106	66	0.002	0.569	0.778

Where: SS effect—sum of the squares of the effect, df effect—number of the degrees of freedom of the effect, MS effect—mean sum of the squares of the effect, SS error—sum of the squares of the error, df error—number of the degrees of freedom of the error, MS error—mean sum of the squares of the error, F—value of tests F, *p*—level of probability *p*.

**Table 2:** Verification of the assumption of the homogeneity of variance by means of the Brown-Forsythe test with sterilization adopted as the grouping variable.

	Brown-Forsythe Variance Homogeneity Test Marked Effects Are Relevant with *p* < 0.05							
Variable	SS Effect	df Effect	MS Effect	SS Error	df Error	MS Error	F	*p*
Strain	0.003	1	0.003	0.112	72	0.002	1.909	0.171

**Table 3:** Results of the multi-factor analysis of variance.

	One-Dimensional Tests of Significance in Relation to Strain Parametrization with Sigma-Limits Decomposition of Effective Hypotheses				
Effect	SS	Degrees of Freedom	MS	F	*p*
Free term	2.449	1	2.449	741.087	0
Reagent	0.017	7	0.002	0.716	0.659
Sterilization	0.0003	1	0.0003	0.104	0.748
Reagent×Sterilization	0.017	7	0.002	0.739	0.631
Error	0.192	58	0.003		

Where: SS—sum of the squares of the effects, MS—mean sum of the squares of the effects, F—value of test F, *p*—level of probability.

**Table 4:** Results of the Kruskal–Wallis test.

Dependent Variable: Young’s Modulus	ANOVA of Kruskal–Wallis Ranks; Young’s Modulus Independent (Grouping) Rank: Reagent Kruskal-Wallis: H (7, N = 36) = 18.83, *p* = 0.0087			
	Code	N Valid	Sum of Ranks	Mean Rank
1	1	4	121	30.25
2	2	4	119	29.75
3	3	4	44	11
4	4	5	96	19.2
5	5	4	84.5	21.13
6	6	5	97.5	19.5
7	7	5	35	7
8	8	5	69	13.8

Where: Code—numerals assigned to individual substances, N valid—number of samples tested within a given group of samples, Sum of ranks—sum of ranks assigned to individual groups, Mean rank—equivalent of the arithmetic mean in parametric tests.

**Table 5:** Results of repeated (multiple) comparisons.

Dependent Variable: Young’s Modulus	Value *p* for Repeated (Multiple) Comparisons; Young’s Modulus Independent (Grouping) Variable: Reagent Kruskal–Wallis Test H (7, N = 36) = 18.83, *p* = 0.087							
	1 R:30.25	2 R:29.75	3 R:11	4 R:19.2	5 R:21.13	6 R:19.5	7 R:7	8 R:13.8
1		1	0.27	1	1	1	0.03	0.56
2	1		0.33	1	1	1	0.04	0.67
3	0.27	0.33		1	1	1	1	1
4	1	1	1		1	1	1	1
5	1	1	1	1		1	1	1
6	1	1	1	1	1		1	1
7	0.03	0.04	1	1	1	1		1
8	0.56	0.67	1	1	1	1	1	

**Table 6:** Results of the Kruskal–Wallis test.

Dependent Variable: Young’s Modulus	ANOVA of Kruskal–Wallis Ranks; Young’s Modulus Independent (Grouping) Rank: Reagent Kruskal–Wallis: H (7, N = 37) = 15.29, *p* = 0.0325			
	Code	N Valid	Sum of Ranks	Mean Rank
1	1	4	96	24
2	2	5	133	26.6
3	3	5	95	19
4	4	5	62	12.4
5	5	4	34	8.5
6	6	5	144	28.8
7	7	5	59	11.8
8	8	4	80	20

**Table 7:** Results of repeated (multiple) comparisons.

Dependent Variable: Young’s Modulus	Value *p* for Repeated (Multiple) Comparisons; Young’s Modulus Independent (Grouping) Variable: Reagent Kruskal–Wallis Test H (7, N = 37) = 15.29, *p* = 0.0325							
	1 R:24	2 R:26.6	3 R:19	4 R:12.4	5 R:8.5	6 R:28.8	7 R:11.8	8 R:20
1		1	1	1	1	1	1	1
2	1		1	1	0.35	1	0.86	1
3	1	1		1	1	1	1	1
4	1	1	1		1	0.46	1	1
5	1	0.35	1	1		0.15	1	1
6	1	1	1	0.46	0.15		0.36	1
7	1	0.86	1	1	1	0.36		1
8	1	1	1	1	1	1	1	
